# Peptidoglycan endopeptidase MepM of uropathogenic *Escherichia coli* contributes to competitive fitness during urinary tract infections

**DOI:** 10.1186/s12866-024-03290-9

**Published:** 2024-05-30

**Authors:** Wen-Chun Huang, Ida Bagus Nyoman Putra Dwija, Masayuki Hashimoto, Jiunn-Jong Wu, Ming-Cheng Wang, Cheng-Yen Kao, Wei-Hung Lin, Shuying Wang, Ching-Hao Teng

**Affiliations:** 1 Institute of Molecular Medicine, College of Medicine, National Cheng Kung University, Tainan, Taiwan; 2 Department of Clinical Microbiology, Faculty of Medicine, Udayana University, Denpasar, Bali, Indonesia; 3 Institute of Basic Medical Sciences, College of Medicine, National Cheng Kung University, Tainan, Taiwan; 4 Department of Medical Laboratory Science and Biotechnology, Asia University, Taichung, Taiwan; 5 Department of Medical Research, China Medical University Hospital, China Medical University, Taichung, Taiwan; 6 Division of Nephrology, Department of Internal Medicine, College of Medicine, National Cheng Kung University Hospital, National Cheng Kung University, Tainan, Taiwan; 7 Institute of Microbiology and Immunology, College of Life Sciences, National Yang Ming Chiao Tung University, Taipei, Taiwan; 8 Department of Internal Medicine, College of Medicine, National Cheng Kung University Hospital, National Cheng Kung University, Tainan, Taiwan; 9 Department of Microbiology and Immunology, College of Medicine, National Cheng Kung University, Tainan, Taiwan

**Keywords:** MepM, YebA, MepS, Spr, Peptidoglycan endopeptidase, Uropathogenic *E. Coli*, UPEC, Urinary tract infection, UTI, Pathogenesis

## Abstract

**Background:**

Urinary tract infections (UTIs) are common bacterial infections, primarily caused by uropathogenic *Escherichia coli* (UPEC), leading to significant health issues and economic burden. Although antibiotics have been effective in treating UPEC infections, the rise of antibiotic-resistant strains hinders their efficacy. Hence, identifying novel bacterial targets for new antimicrobial approaches is crucial. Bacterial factors required for maintaining the full virulence of UPEC are the potential target. MepM, an endopeptidase in *E. coli*, is involved in the biogenesis of peptidoglycan, a major structure of bacterial envelope. Given that the bacterial envelope confronts the hostile host environment during infections, MepM’s function could be crucial for UPEC’s virulence. This study aims to explore the role of MepM in UPEC pathogenesis.

**Results:**

MepM deficiency significantly impacted UPEC’s survival in urine and within macrophages. Moreover, the deficiency hindered the bacillary-to-filamentous shape switch which is known for aiding UPEC in evading phagocytosis during infections. Additionally, UPEC motility was downregulated due to MepM deficiency. As a result, the *mepM* mutant displayed notably reduced fitness in causing UTIs in the mouse model compared to wild-type UPEC.

**Conclusions:**

This study provides the first evidence of the vital role of peptidoglycan endopeptidase MepM in UPEC’s full virulence for causing UTIs. MepM’s contribution to UPEC pathogenesis may stem from its critical role in maintaining the ability to resist urine- and immune cell-mediated killing, facilitating the morphological switch, and sustaining motility. Thus, MepM is a promising candidate target for novel antimicrobial strategies.

**Supplementary Information:**

The online version contains supplementary material available at 10.1186/s12866-024-03290-9.

## Background

Uropathogenic *Escherichia coli* (UPEC) is the leading cause of urinary tract infections (UTIs), accounting for 75% of community-acquired UTIs and 65% of hospital-acquired UTIs [1, 2]. The majority of bacterial UTIs occur via an ascending pathway. UPEC first enter the urinary tract through the urinary meatus, then ascend through the urethra and establish colonization in the bladder, leading to cystitis. In some cases, the pathogens can progress further up the ureters and reach the kidneys, resulting in pyelonephritis. In some severe cases of pyelonephritis, UPEC can cross the tubular epithelial cell barrier to enter the bloodstream to progress to bacteremia [3]. Although antibiotics are commonly used to treat bacterial infections, the emergence of antibiotic-resistant strains poses a significant public health threat. Therefore, it is essential to identify and investigate new antibiotic targets to develop effective antimicrobial strategies and combat this public health crisis [3]. Bacterial factors that are required for maintaining the whole virulence of UPEC are the potential antimicrobial targets [4].

Peptidoglycan (PG), which is composed of glycan strands cross-linked by short peptide chains, is a vital component of the bacterial cell wall, crucial for maintaining bacterial morphology and protecting against environmental stresses [5]. Disrupting the biogenesis of peptidoglycan can interfere with bacterial growth and impair bacterial virulence [6, 7]. As bacteria grow, the peptidoglycan sacculus must expand by breaking old peptide crosslinks followed by incorporating newly synthesized glycan strands into the primary PG structures [8]. Accordingly, the enzymes mediating the new glycan strand synthesis and the one mediating the crosslink breakage are both required for maintaining the intact structure of PG. Inactivation of the enzymes mediating the glycan synthesis is well known to blocks bacterial growth and thus has long been served as effective targets of antibiotics, such as β-lactams [7]. However, the impact of inactivating the enzymes, specifically the endopeptidases, involved in the peptide crosslink breakage on bacteria remains to be investigated.

In our previous study, we demonstrated the essential role of MepS (also known as Spr), one of the peptidoglycan D, D-endopeptidases, in maintaining the intact virulence of UPEC [6]. Alongside MepS, two other D, D-endopeptidases, namely MepM (also known as YebA) and MepH (also known as YdhO), have been reported to be functionally redundant in supporting *E. coli*’s survival [9]. Recent research has made progress in identifying the distinct characteristics of MepM and MepS in *E. coli*’s resistance to environmental stresses. Deletion of *mepM* decreases bacterial resistance to salt stress, while deletion of *mepS* reduces resistance to EDTA stress [10]. These findings suggest that these endopeptidases have both common and unique roles in combating different environmental stresses and thus likely in the pathogenesis of UPEC. Since the exact role of MepM in the pathogenesis of UPEC remains unclear, this study aimed to evaluate the specific role of MepM in maintaining the intact virulence of UPEC.

In order to establish infections, UPEC must possess specific virulence features to overcome the challenging conditions within the urinary tract. These environmental challenges include limited availability of iron nutrients within the host, the antimicrobial nature of urine, and the mechanical flushing caused by urine flow [11, 12]. UPEC also face attacks from the host’s immune system during infection [3]. Additionally, motility and the ability to transition from a bacillary to a filamentous morphology have been recognized as crucial UPEC features that contribute to UTIs [13, 14]. Flagellum-mediated motility enables UPEC to effectively colonize the urinary tract and disseminate within the host [13]. The morphological switch to a filamentous shape during UTIs allows UPEC to evade phagocytosis and thus escape the killing of phagocytes [14]. Since the bacterial envelope of UPEC is where the bacteria interact with the harsh host environment, we investigated whether MepM contributes to the virulence features of UPEC.

## Results

### Deletion of *mepM* does not result in growth defects in UPEC strain UTI89

**Fig. 1:**
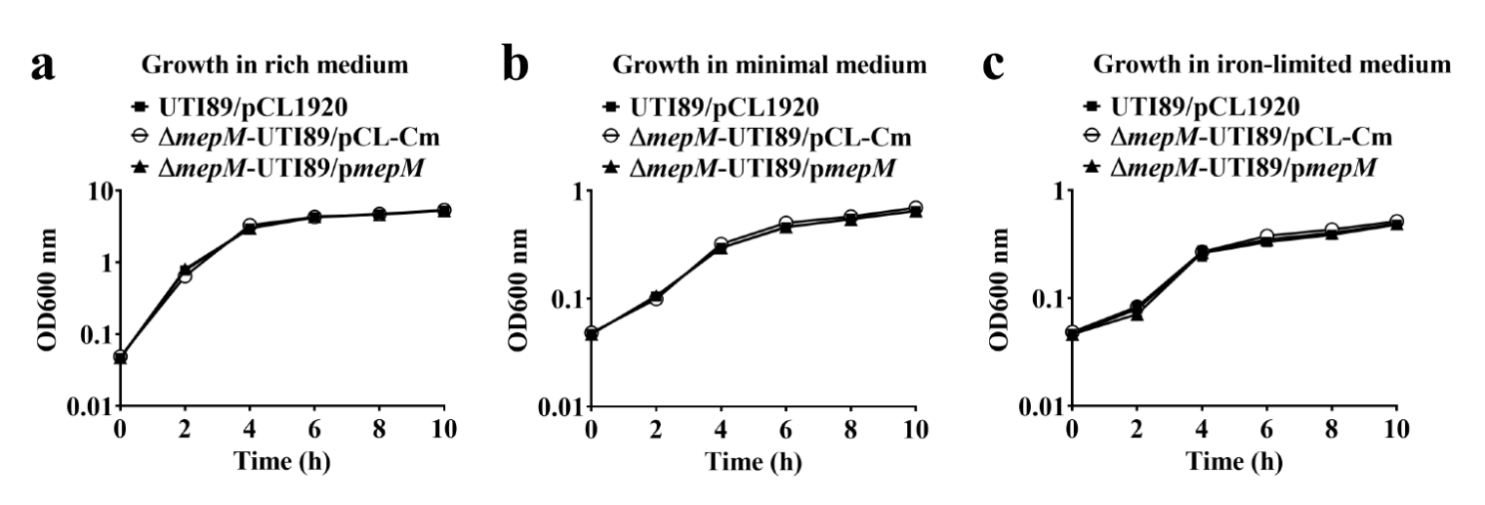
The growth of the wild type UTI89, *mepM* mutant, and trans-complemented strains under different conditions. (**a**) The growth of the bacteria in LB rich medium. (**b**) The growth of the bacteria in M9 minimal medium. (**c**) The growth of the bacteria in iron-limited medium.UTI89/pCL1920 is the wild type UPEC strain UTI89 harboring the empty vector pCL1920. Δ*mepM*-UTI89/p*mepM* is the Δ*mepM*-UTI89 strain harboring the plasmid encoding MepM, while Δ*mepM*-UTI89/pCL-Cm is the mutant strain harboring the plasmid pCL-Cm, which is the empty vector pCL1920 harboring a chloramphenicol resistance cassette instead of the *mepM* gene (Table 1)

To investigate whether the PG DD-endopeptidase, MepM (YebA) involves the intact virulence of UPEC during UTIs, the wild type UPEC strain UTI89 harboring empty vector pCL1920 (UTI89/pCL1920), the *mepM* deletion mutant harboring the otherwise empty vector pCL-Cm (Δ*mepM*-UTI89/pCL-Cm), and the trans-complemented strain (Δ*mepM*-UTI89/p*mepM*) were constructed (Table 1) and their growth under different conditions were determined. The *mepM* mutant showed no significant difference in growing at rich (LB), minimal (M9), and iron-limited media in comparison with the wild-type UTI89 (UTI89/pCL1920) and the trans-complemented strain (Δ*mepM*-UTI89/p*mepM*) (Fig. 1a–c). These findings suggest that UTI89 strain lacking DD-endopeptidase MepM does not impair bacterial growth under rich (LB), minimal (M9), and iron-limited media.

**Table 1:** *E.coli* strains and plasmids used in this study

Strain or Plasmid	Relevant information	AR marker^*^	Reference
**Strains**			
UTI89	UTI89 (serotype O18:K1:H7) isolated from the urine of a patient with cystitis	-	[42]
Δ*mepM*-UTI89	UTI89 with a *mepM* deletion	Km	This study
**Plasmids**			
pCL1920	Low copy number plasmid vector	Sp	[24]
pCL-Cm	pCL1920 harboring a chloramphenicol resistance cassette	Cm, Sp	This study
p*mepM*	pCL1920 harboring the *mepM* gene which is under control of the *lac* promoter on the plasmid	Sp	This study
pCL-FlhDC	pCL1920 harboring a sequence encoding the N-terminally HA-tagged FlhD and C-terminally His_6_-tagged FlhC that are under control of *lac* promoter	Sp	This study
pFPV25.1	GFP expressing plasmid	Amp	[43]
pmCherry	The pFPV25.1 plasmid on which the GFP-encoding sequence is replace with a mCherry-encoding sequence	Amp	This study

^*^AR marker, antibiotic resistance marker: Km, Kanamycin; Sp, Spectinomycin; Cm, Chloramphenicol; Amp, Ampicillin

### *mepM* deletion decreases ability of UPEC to resist immune cell-mediated killing

During UTIs, UPEC interact with host cells. For instance, UPEC needs to adhere to uroepitheliums to establish colonization in the urinary tract. Additionally, the pathogens may encounter and be phagocytosed by immune cells during the infection process. To investigate whether deletion of *mepM* affect the abilities of UPEC to interact with host cells, the abilities of Δ*mepM*-UTI89 to associate to bladder epithelial cells and to survive within immune cells were evaluated.

**Fig. 2:**
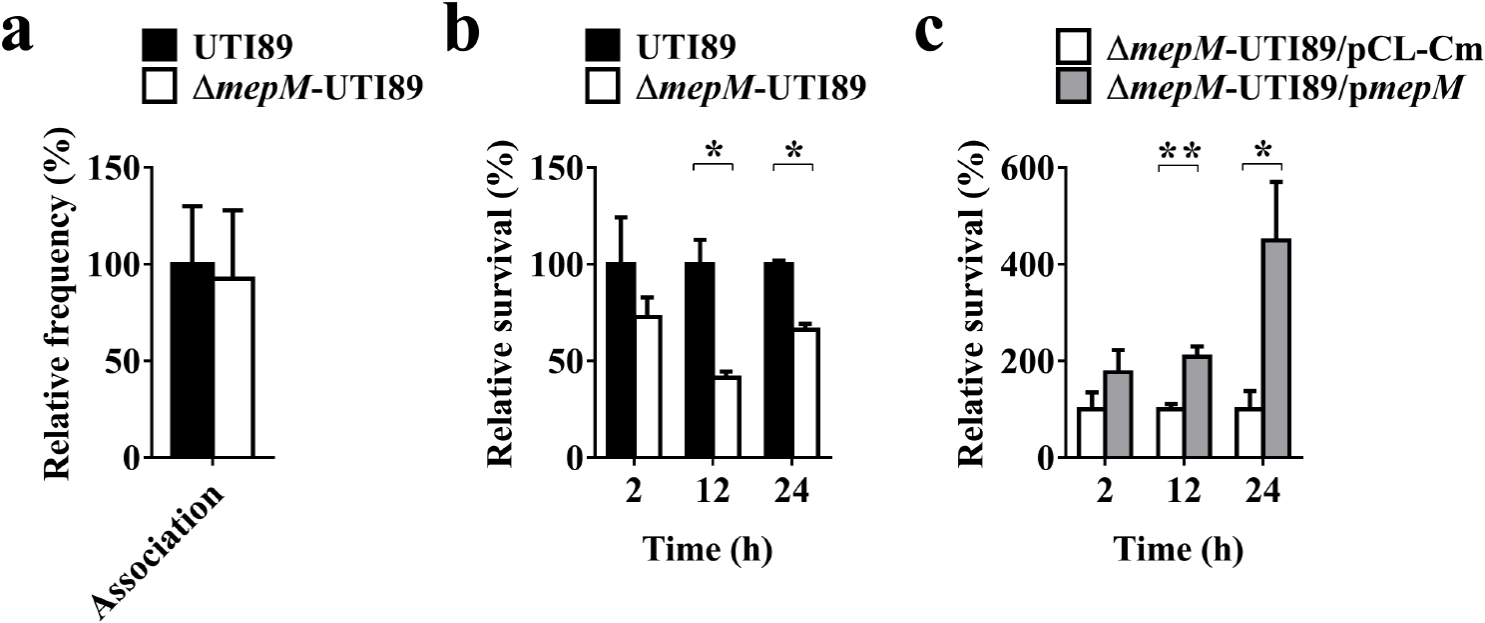
The abilities of Δ*mepM*-UTI89 to interact with host cells. (**a**) The abilities of Δ*mepM*-UTI89 and UTI89 to associate to the bladder epithelial cell 5637. (**b**) The intracellular survival of Δ*mepM*-UTI89 and UTI89 within the macrophage cell RAW264.7. (**c**) The intracellular survival of Δ*mepM*-UTI89/p*mepM* and Δ*mepM*-UTI89/pCL-Cm with the macrophage cell RAW264.7. The association and survival levels were shown as the percentages relative to those of UTI89. The data shown are representative of three independent experiments which were performed in triplicate. The results are shown as the mean ± standard deviations. *, *P* < 0.05; **, *P* < 0.01

To investigate the interaction with uroepitheliums, UTI89 and Δ*mepM*-UTI89 strains were mixed in equal numbers and incubated with the bladder epithelial cell 5637. After a 90-minute incubation, the bacteria associated to the cells were evaluated. As shown in Fig. 2a, both strains exhibited a similar level of ability to associate to the 5637 cells. Consistently, in comparison with the wild-type UTI89 strain, the *mepM* mutant showed no significant difference in the transcript levels of fimbriae-associated genes (Fig. S1), which have been shown to be involved in interaction with epithelial cells [15]. These findings suggest that MepM deficiency does not significantly affect UPEC’s ability to associate to uroepitheliums.

To study the interaction with immune cells, equal amounts of UTI89 and Δ*mepM*-UTI89 were co-inoculated into the culture of the macrophage cell RAW264.7. After a 30-minute incubation to facilitate bacterial phagocytosis by macrophages, gentamicin treatment was applied to eliminate bacteria not internalized by the cells, and subsequently, the survival of the internalized bacteria was assessed. Δ*mepM*-UTI89 exhibited significantly lower survival rates than UTI89 within the macrophages (Fig. 2b). Consistently, when Δ*mepM*-UTI89 was trans-complemented with *mepM*, the survival rates of the mutant increased (Fig. 2c). These findings suggest that MepM is required for the intact ability of UPEC to survive in immune cells.

As pathogens phagocytosed by immune cells encounter a low-pH environment in the phagosome, we investigated whether MepM deficiency decreases UPEC’s survival in such conditions. However, our results revealed that Δ*mepM*-UTI89 and UTI89 exhibited similar survival rates at pH 4.5 in LB with 2 h incubation (data not shown), indicating that MepM deficiency does not influence UPEC’s resistance to a low-pH environment. In addition, K1 capsule of *E. coli* prevents phagosome-lysosome fusion that leads to lysis of the internalized bacterial cells [16]. Thus, we examined the transcript levels of K1 capsule-producing genes. As shown in Fig. S1, Δ*mepM*-UTI89 and UTI89 exhibited similar levels of K1 capsule-associated genes. Consistently, no significant difference of capsule production was observed between UTI89 and Δ*mepM*-UTI89 (Fig. S2). These results suggested that deletion of *mepM* might not influence the phagosome-lysosome fusion that mediated by K1 capsule expression.

### *mepM* deletion decreases the morphology switch of UPEC during interaction with bladder epithelial cells in vitro

**Fig. 3:**
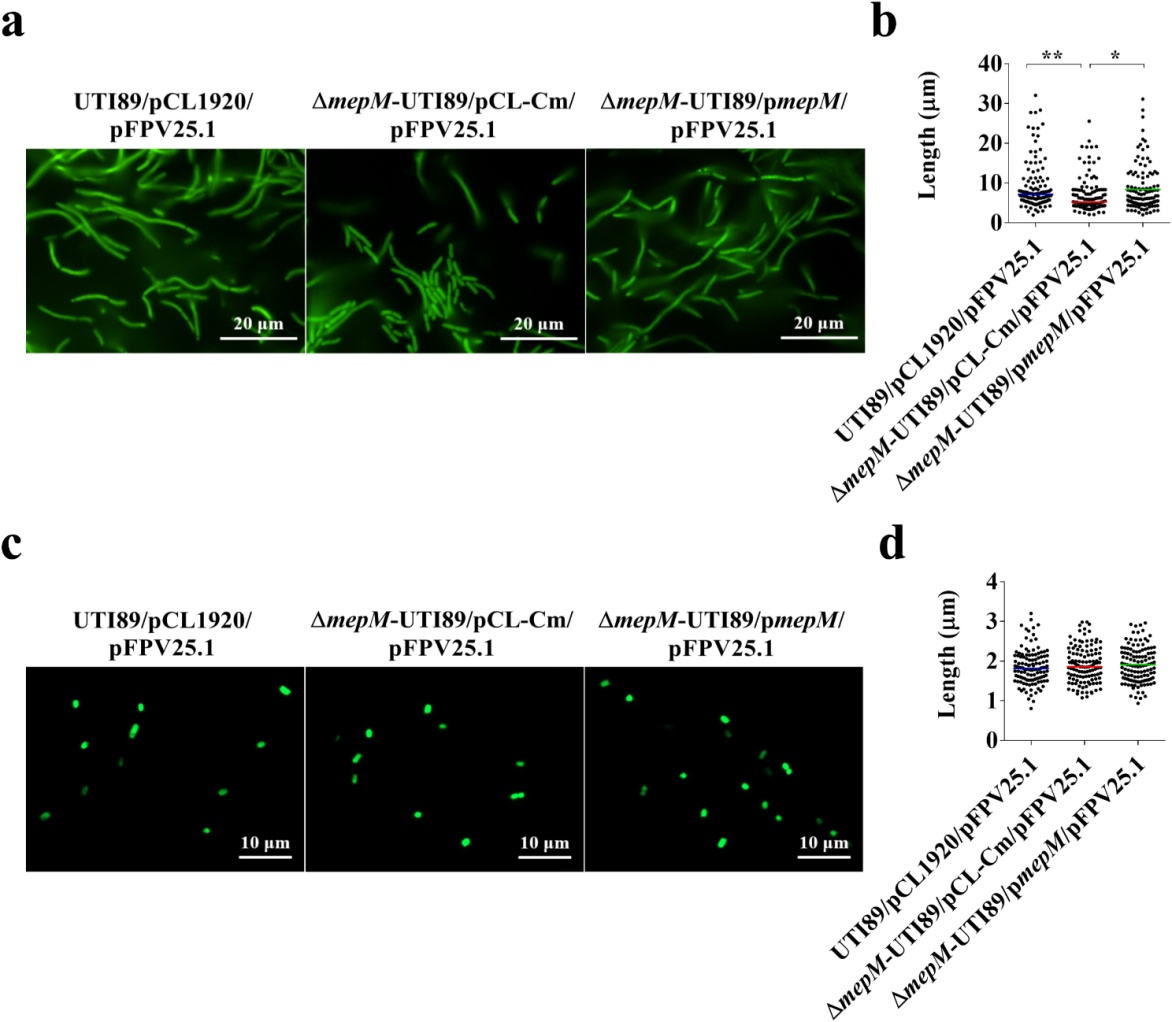
Morphological comparison of the wild type UTI89, *mepM* mutant, and trans-complemented strains in the in vitro FC-based infection model and LB Medium. (**a**) Fluorescence microscopy images of the UTI89/pCL1920, Δ*mepM*-UTI89/pCL-Cm, and Δ*mepM*-UTI89/p*mepM* strains carrying pFPV25.1 (UTI89/pCL1920/pFPV25.1, Δ*mepM*-UTI89/pCL-Cm/pFPV25.1, and Δ*mepM*-UTI89/p*mepM*/pFPV25.1; Table 1) after incubation with bladder epithelial cells in the in vitro FC-based infection model using human urine as medium. (**b**) Quantitative analysis of bacterial length following incubation in the FC-based infection model. (**c**) Fluorescence microscopy images of UTI89/pCL1920/pFPV25.1, Δ*mepM*-UTI89/pCL-Cm/pFPV25.1, and Δ*mepM*-UTI89/p*mepM*/pFPV25.1 cultured in LB medium. (**d**) Quantitative analysis of bacterial length after incubation in LB medium. For bacterial length quantification, fluorescence microscopy images were analyzed using ImageJ (National Institutes of Health; Bethesda, MD, United States). The length of 120 bacterial cells from three microscopic fields (40 cells/field) was measured. The horizontal bars indicate the median bacterial size. ****, *P* < 0.001

It is known that filamentous morphology provides UPEC with an advantage to resist phagocytosis [14]. We investigated the impact of MepM deficiency on the morphological switch of UPEC from bacillary to filamentous form after interacting with bladder epitheliums. This investigation utilized an in vitro model of human bladder cell infection, employing a FC-based culture system with human urine. This system closely mimics the in vivo conditions and enables the observation of UPEC’s morphological transition [6, 17] (see Methods). We separately incubated the wild type UTI89, *mepM* mutant, and trans-complemented strains that harbor the GFP-encoding plasmid pFPV25.1 (UTI89/pCL1920/pFPV25.1, Δ*mepM*-UTI89/pCL-Cm/pFPV25.1, and Δ*mepM*-UTI89/p*mepM*/pFPV25.1; Table 1) with the 5637 cells in the culture system. Then, the morphology of the bacteria was investigated by fluorescence microscopy. Elongated filamentous bacteria were found in these three strains (Fig. 3a). However, the *mepM* mutant cells showed a significantly lower level of elongation than the wild type UTI89 cells and the trans-complemented cells (Fig. 3b). On the other hand, in the LB medium, the three strains showed similar bacterial lengths (Fig. 3c, d). These findings suggest that MepM deficiency may attenuate the morphological switch of UPEC in UTIs.

### *mepM* deletion decreases the motility of UPEC

**Fig. 4:**
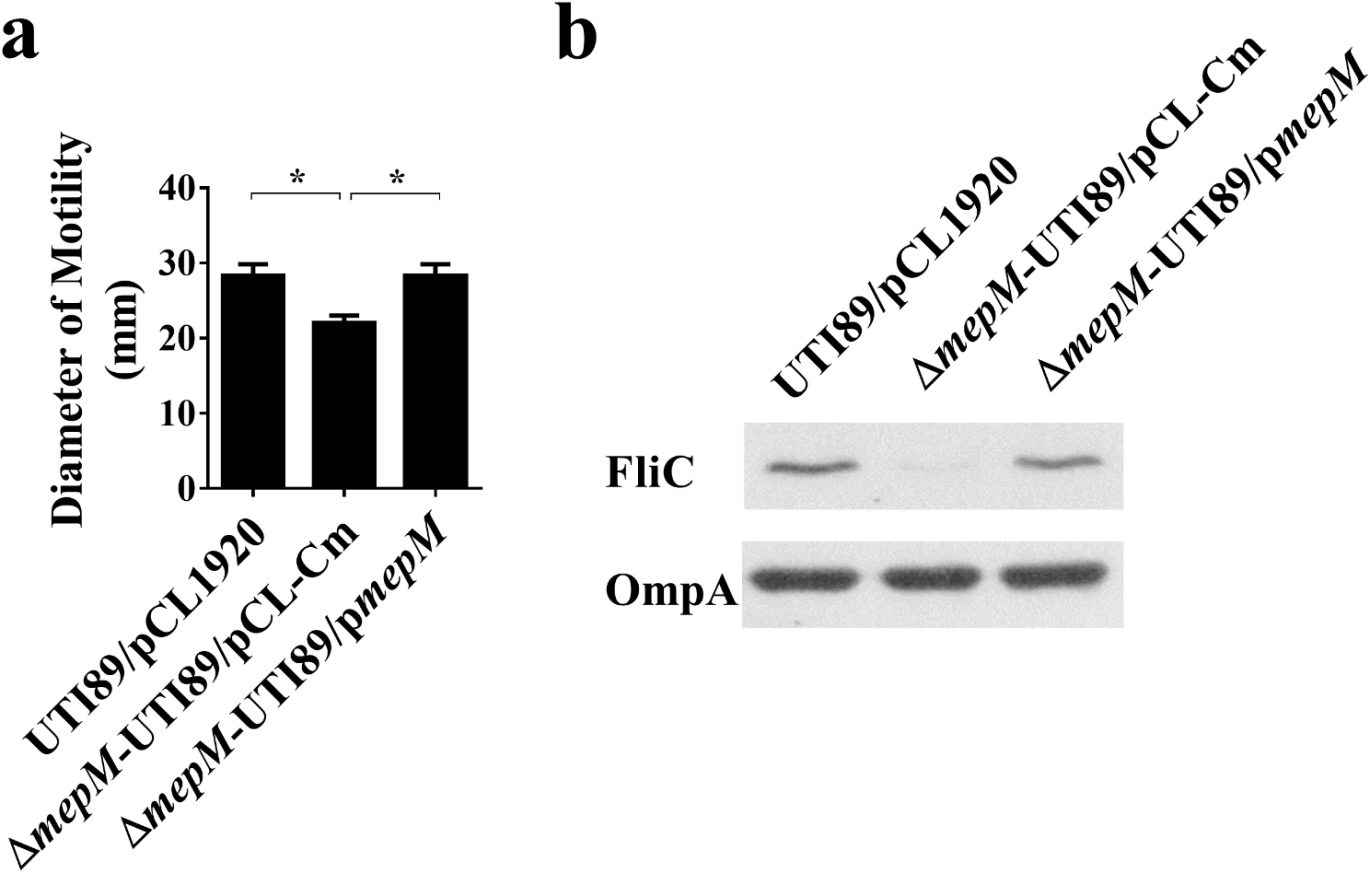
The impacts of *mepM* deletion on the motility and flagellin expression of UPEC. (**a**) The motility of UTI89/pCL1920, Δ*mepM*-UTI89/pCL-Cm, and Δ*mepM*-UTI89/p*mepM*. (**b**) Western blot analysis of FliC (flagellin) levels in the UTI89 strains. The levels of FliC were determined using rabbit antiserum against FliC. The levels of the outer membrane proteins A (OmpA) of *E. coli* was used as a protein loading control, which were determined using a mouse anti-OmpA serum. The quantified motility of (**a**) UTI89/pCL1920, Δ*mepM*-UTI89/pCL-Cm, and Δ*mepM*-UTI89/p*mepM* were derived from experiments performed in triplicate and is presented as the means ± standard deviations. *, *P* < 0.05. The full-length blots are shown in Fig. S1

Given that bacterial motility confers fitness to UPEC during UTIs [18, 19], we further investigated whether deletion of *mepM* affects the motility of UPEC. As shown in Fig. 4a, deletion of *mepM* resulted in a 22% decrease in motility. Additionally, trans-complementation of Δ*mepM*-UTI89 with *mepM* apparently increased the bacterial motility (Fig. 4a). In agreement with these findings, the levels of the major flagellum component protein FliC (flagellin) in Δ*mepM*-UTI89 was lower than that in UTI89 (Figs. 4b and S3). Our findings suggest that deletion of *mepM* downregulates flagella expression, consequently decreasing bacterial motility.

### Deletion of *mepM* decreases the fitness of UPEC in mouse model of UTIs

**Fig. 5:**
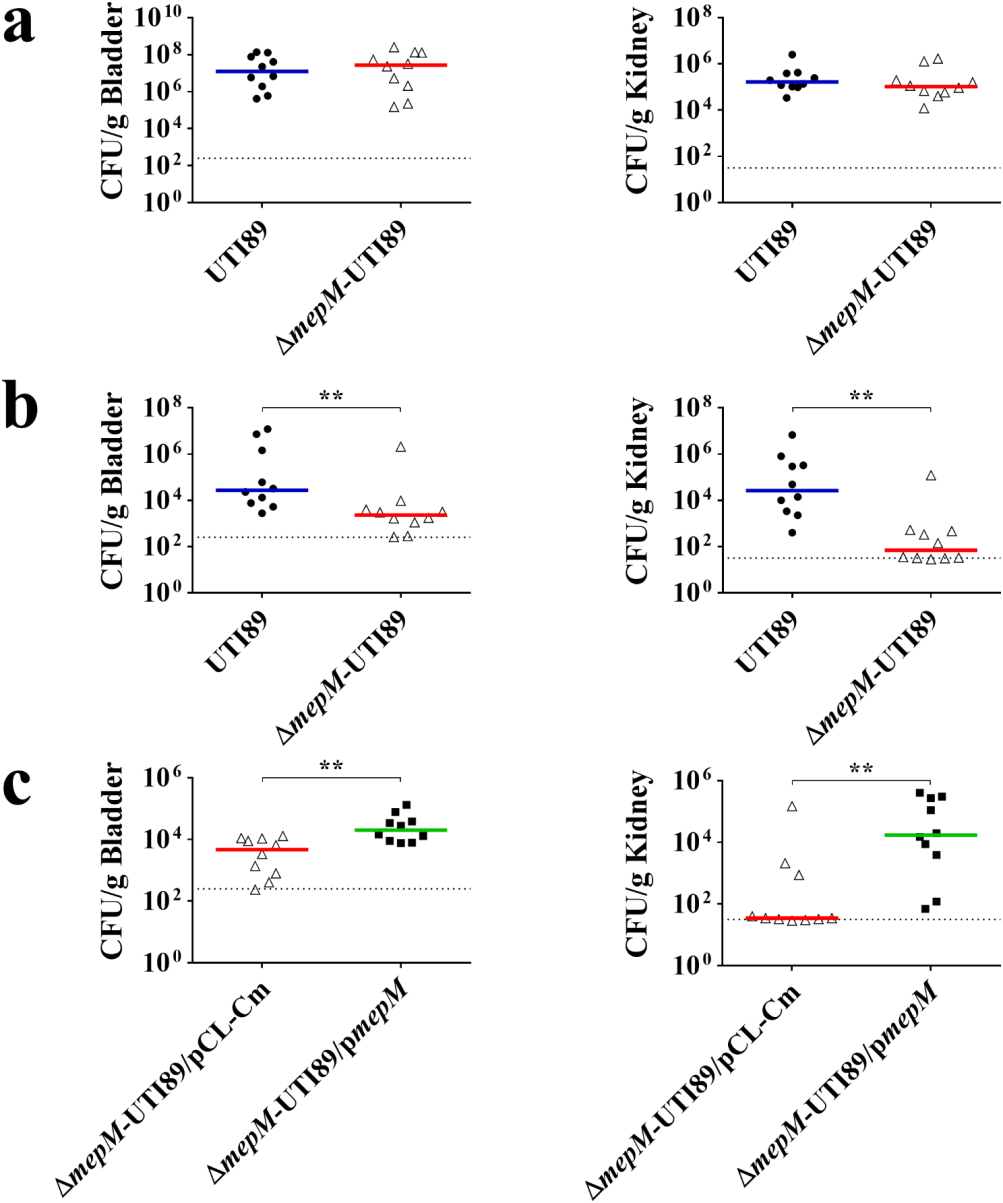
Impact of *mepM* deletion on UPEC’s ability to cause UTIs. (**a**) Bacterial counts of UTI89 and Δ*mepM*-UTI89 in the bladders and kidneys at 2 days after transurethral co-inoculation into mice. (**b**) Bacterial counts of UTI89 and Δ*mepM*-UTI89 in the organs at 14 days after transurethral co-inoculation of the strains into animals. (**c**) Bacterial counts of Δ*mepM*-UTI89/pCL-Cm and Δ*mepM*-UTI89/p*mepM* in the bladders and kidneys at 14 days after transurethral co-infection. Each co-infection involved 5 × 10^7^ CFU/strain/mouse of bacteria, and 10 animals (*N* = 10) were used. The horizontal bars represent the median values, and the dotted line indicates the limit of detection. **, *P* < 0.01

To investigate whether *mepM* deficiency interferes with the intact virulence of UPEC, Δ*mepM*-UTI89 and UTI89 were subjected to a mouse model of UTIs. Equal amounts of the strains were transurethrally co-inoculated into animals. At 2 days and 14 days post-infection, the bacterial burdens in the bladders, kidneys and blood were evaluated. Although at 2 days post-infection the counts of the two strains in the bladders and kidneys showed no significant difference (Fig. 5a), at 14 days post-infection Δ*mepM*-UTI89 showed significantly lower bacterial counts than UTI89 in both organs (Fig. 5b). Complementation with the *mepM* gene significantly increased the *mepM* mutant’s counts in bladders and kidneys (Fig. 5c). In addition, there were no recovered bacteria from blood at 2 days and 14 days post-infection (data not shown). These findings demonstrate that MepM is required for the full fitness of UPEC during UTIs.

### *mepM* deletion decreases ability of UPEC to the growth fitness in the mouse urine

**Fig. 6:**
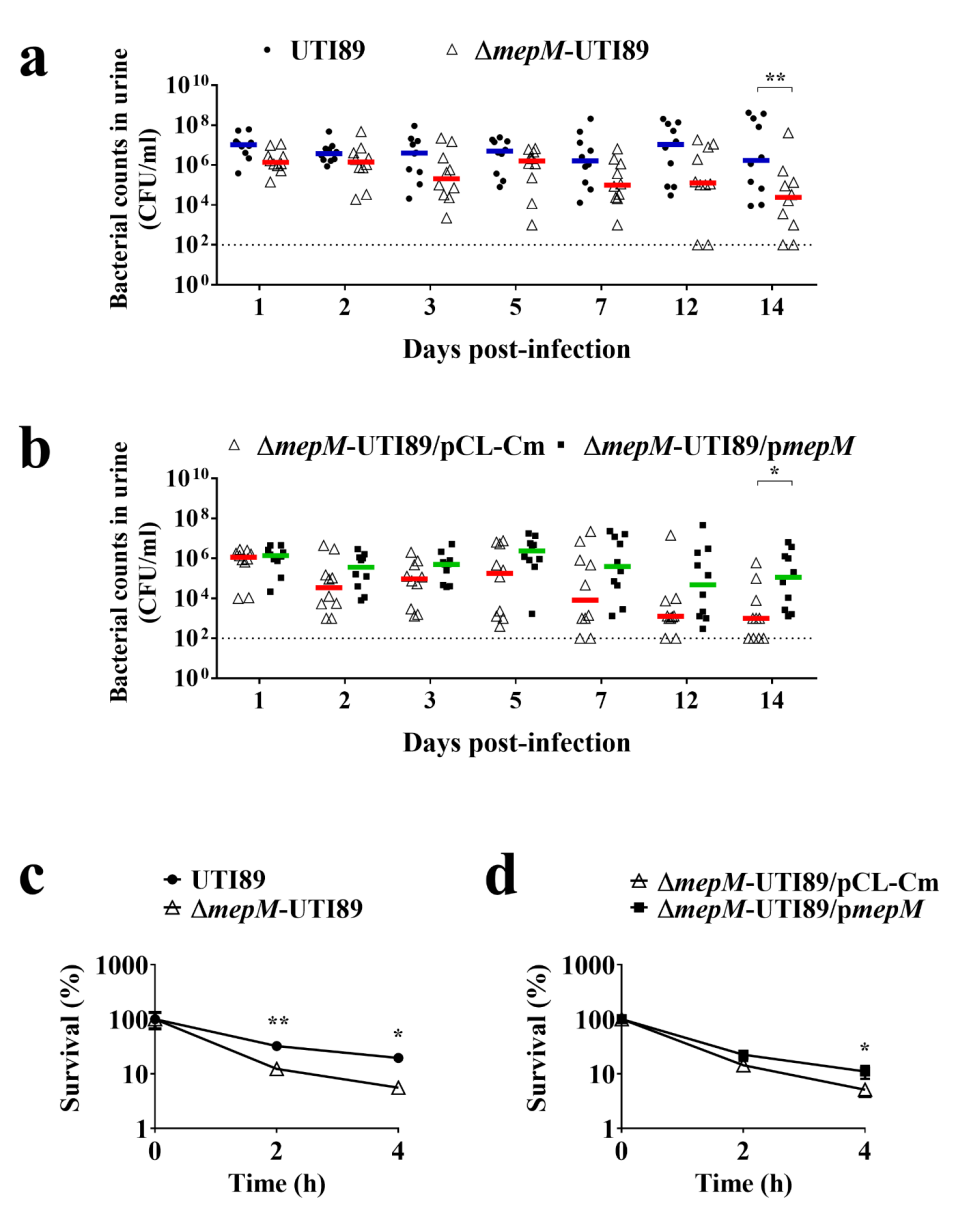
Impact of *mepM* deletion on UPEC’s ability to survive in urine. (**a**) Bacterial counts of UTI89 and Δ*mepM*-UTI89 in the infected mouse urine collected at the indicated time after transurethral co-infection. (**b**) Bacterial counts of Δ*mepM*-UTI89/pCL-Cm and Δ*mepM*-UTI89/p*mepM* in the infected mouse urine collected at the indicated time after transurethral co-infection. (**c**) The survival of the Δ*mepM*-UTI89 and UTI89 co-cultured in pooled mouse urine. The relative survival were the relative survival bacterial counts related to the inoculum. (**d**) The survival of the Δ*mepM*-UTI89 strains with or without trans-complementation of *mepM* co-cultured in the mouse urine. For (**a**) and (**b**), each co-infection involved 5 × 10^7^ CFU/strain/mouse of bacteria, and 10 animals (*N* = 10) were used. The horizontal bars represent the median values, and the dotted line indicates the limit of detection. For (**c**) and (**d**), the data shown are representative of three independent experiments which were performed in triplicate. The results are shown as the mean ± standard deviations. *, *P* < 0.05; **, *P* < 0.01

Consistent with the organs from the infected mice, in the urine of the infected animals, the levels of the Δ*mepM*-UTI89 was significantly outcompeted by UTI89 at 14 days post-infection (Fig. 6a). Trans-complementation with the *mepM* gene significantly increased the *mepM* mutant’s counts in urine (Fig. 6b).

We further investigated whether deletion of *mepM* affects the ability of UPEC to survive in the mouse urine. Equal amounts of Δ*mepM*-UTI89 and UTI89 were co-inoculated into pooled mouse urine and the relative bacterial levels were determined at indicated time points. As shown in Fig. 6c, the survival level of UTI89 were significantly higher than that of Δ*mepM*-UTI89 after 2 h and 4 h of incubation. Consistently, trans-complementation of the mutant with *mepM* significantly increased the bacterial survival in the urine co-culture experiments (Fig. 6d). However, the *mepM* mutant showed no significant difference in growing in pooled healthy human urine in comparison with the wild-type UTI89 (data not shown). These findings suggest that MepM is required for the intact ability of UPEC to survive in the mouse urine.

### The decreased motility contributes to the fitness defect caused by MepM deficiency

**Fig. 7:**
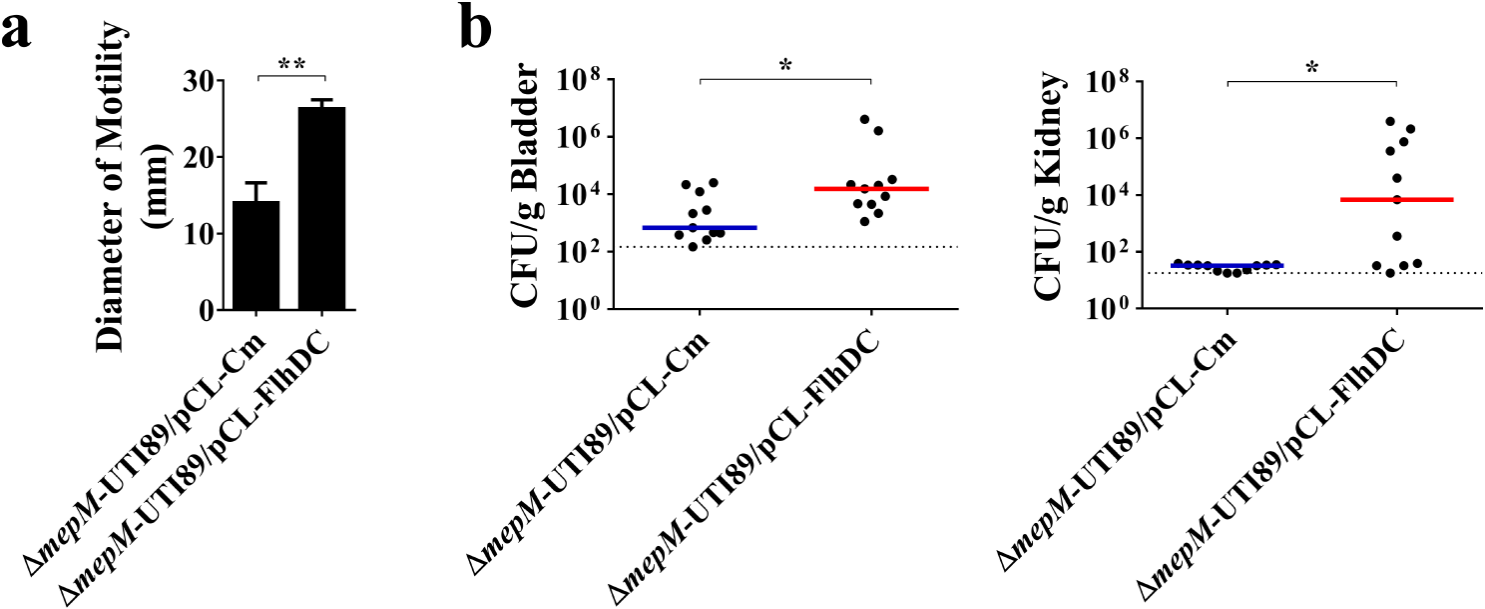
Impact of bacterial motility on the Δ*mepM*-UTI89’s ability to cause UTIs. (**a**) Motilities of UTI89 strains with and without FlhDC overexpression. Each result represents the mean ± standard deviation from triplicate experiments. (**b**) Bacterial counts of Δ*mepM*-UTI89 strains with and without FlhDC overexpression in the bladders and kidneys at 14 days after co-inoculation into mice. The experiments involved 11 animals (*N* = 11). The horizontal bars indicate the median values, and the dotted line represents the limit of detection. *, *P* < 0.05; **, *P* < 0.01

Since deletion of *mepM* decreased the motility of UPEC, we further determined whether the impaired motility of Δ*mepM*-UTI89 contributes to its defected fitness during UTIs. We overexpressed the master transcriptional regulator, FlhDC, of flagellum regulon in the mutant by introducing the FlhDC-encoding plasmid, pCL-FlhDC (Δ*mepM*-UTI89/pCL-FlhDC; Table 1) [20]. As shown in Fig. 7a, Δ*mepM*-UTI89/pCL-FlhDC showed a higher level of motility than Δ*mepM*-UTI89 harboring the empty plasmid vector pCL-Cm (Δ*mepM*-UTI89/pCL-Cm; Table 1), suggesting that overexpressing FlhDC increases the motility of Δ*mepM*-UTI89. Equal amounts of Δ*mepM*-UTI89/pCL-FlhDC and Δ*mepM*-UTI89/pCL-Cm were mixed and subjected to the mouse UTI model. At 14 days post-infection, the amounts of Δ*mepM*-UTI89/pCL-FlhDC were significantly higher than those of Δ*mepM*-UTI89/pCL-Cm in bladders and kidneys (Fig. 7b). These findings suggest that increasing the motility of the *mepM* mutant can upregulate its fitness in UTIs, and thus indicate that the impaired motility of the mutant contributes to its fitness defect.

### The *mepM* mutant of UPEC shows a lower level of filamentous morphology switch during UTIs

**Fig. 8:**
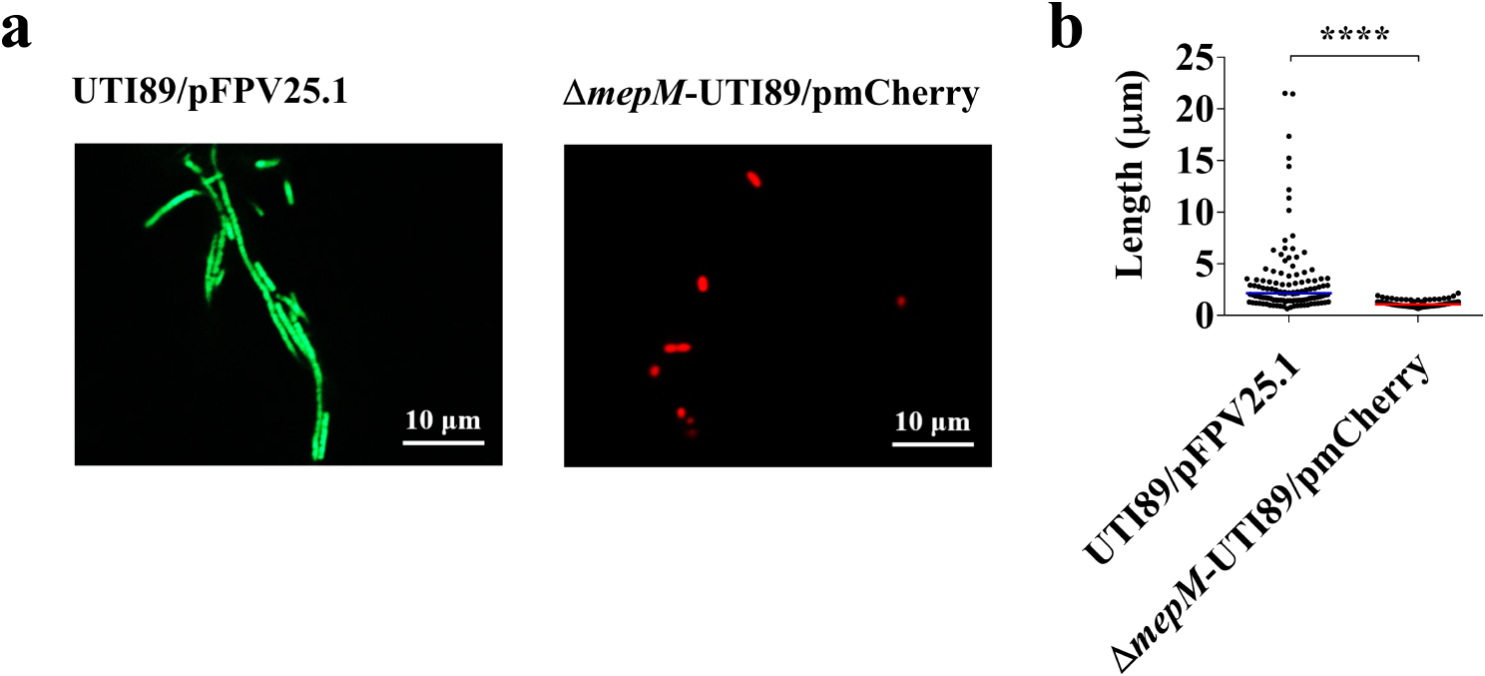
Morphology of UTI89 strains with and without *mepM* in the mouse model of UTIs. (**a**) Fluorescence microscopy images of UTI89/pFPV25.1 and Δ*mepM*-UTI89/pmCherry in the urine of the mice infected with the respective bacterial strains. The urine samples were collected from the animals at 5 days post-infection (**b**) Size quantification of UTI89/pFPV25.1 or Δ*mepM*-UTI89/pmCherry in the urine samples. For each bacterial strain, the sizes of 120 bacteria in the urine samples from three infected animals (40 bacteria/ animal) were determined by imageJ analysis with the fluorescence microscopy images and included in the quantitative results. The horizontal bars indicate the median of the bacterial sizes. ****, *P* < 0.001

To further determine the impact of MepM deficiency on the filamentous morphology switching of UPEC during UTIs in vivo, we separately inoculated UTI89/pFPV25.1 and Δ*mepM*-UTI89/pmCherry strains into mice via the urinary tract. At 5 days post-infection, urine samples were collected from the infected animals and analyzed using fluorescence microscopy (Fig. 8a). Δ*mepM*-UTI89/pmCherry cells showed significantly shorter lengths compared to UTI89/pFPV25.1 (Fig. 8b). These findings clearly demonstrate that MepM deficiency reduces UPEC’s ability to undergo filamentous morphology switching during UTIs in vivo.

## Discussion

This study provides the first evidence that the endopeptidase MepM plays a substantial role in maintaining the intact virulence of UPEC during UTIs. The absence of MepM significantly impeded UPEC’s survival fitness in urine and within immune cells. Furthermore, the deficiency of MepM led to a decrease in flagellum expression, resulting in reduced motility, and hindered transition of UPEC to filamentous morphology. Notably, MepM deficiency impaired competitive fitness of UPEC in a mouse model of UTIs.

Deficiency of MepM may impair the virulence features of UPEC by compromising the integrity of the bacterial envelope, which is primarily consist of the outer membrane (OM), peptidoglycan, and inner membrane (IM). The bacterial envelope serves as a protective barrier against the harsh host environment. Our previous study has shown that deficiency of the peptidoglycan peptidase MepS leads to significant alterations in the levels of OM and IM proteins [6], indicating that peptidoglycan endopeptidase deficiency compromises envelope integrity. Considering that MepM shares the same endopeptidase function as MepS [9], it is reasonable to speculate that the absence of MepM may also alter the envelope components, leading to impaired integrity. The observation of reduced flagellum expression in the Δ*mepM*-UTI89 strain (Fig. 4b) may support this hypothesis. It is known that flagellum expression is negatively regulated by the activation of extracytoplasmic stress systems, such as two-component transduction systems, which are triggered by detecting envelope damage [21]. Therefore, the decreased flagellum expression in the *mepM* mutant is likely a consequence of potential envelope impairment caused by MepM deficiency.

When evaluating the contribution of *mepM* to *E. coli*’s ability to survive within macrophages (Fig. 2b, c), we noticed that, in comparison to strains lacking the gene (Δ*mepM*-UTI89/pCL-Cm or Δ*mepM*-UTI89), the trans-complemented strain (Δ*mepM*-UTI89/p*mepM*) exhibited enhanced relative intracellular survival compared to the wild-type. The expression levels of *mepM* in the trans-complemented may be different form that in the wild-type strain, because the copy numbers and the driving promoter of the *mepM* gene in the two strains are different. This distinction could contribute to the observed discrepancy in the intracellular survival of the two strains. It is has been known that the expression of *mepM* is upregulated when under oxidative stress [22], which is a type of stress the invading bacteria usually encounter within macrophages [23]. Higher levels of *mepM* expression may facilitate *E. coli* resistance to the stress and thus the bacterial survival within immune cells. The level of *mepM* expression in the trans-complemented strain is likely higher than that of the wild-type strain, because the trans-complemented strain harbors higher copy numbers of *mepM* than the wild-type strain. In the trans-complemented strain the *mepM* gene was carried by the plasmid pCL1920 that presents at copy numbers of 5 in bacterial cells [24], while in the wild-type strain only one copy of the gene is encoded in the chromosome. Additionally, the *mepM* encoded in pCL1920 is driven by a *lac* promoter in the complemented strain and the *mepM* gene in the wild-wild type was driven by its original promoter. Although we did not intentionally induce the overexpression of the *lac* promoter-driven *mepM* in the trans-complemented strain during the experiments, the leakage of the *lac* promoter may still result in higher levels of *mepM* expression than its original promoter, thereby causing an elevated expression level in the trans-complemented strain.

The impact of MepM endopeptidase deficiency on the pathogenic properties of UPEC shares similarities with the effects observed in the absence of another endopeptidase, MepS, but also exhibits distinct characteristics of its own [6]. Deletions of either *mepM* or *mepS* reduced the fitness of intracellular survival in immune cells, flagellum-mediated motility, and morphological switch during UTIs. However, MepS-deficient UPEC showed a decreased ability to survive under low-pH conditions [6], whereas the MepM-deficient strain did not exhibit this trait (data not shown). Additionally, both endopeptidases contributes to the competitive fitness in colonizing the bladders and kidneys, but their contributions is located in difference infection stages in the mouse model of UTIs. The contribution of MepM was significantly observed at 14 days post-infections, while that of MepS was observed at 2 days post-infection [6]. These findings suggest that the contributions of the two endopeptidase to the intact virulence are partly distinct.

The different effects of MepM deficiency and MepS deficiency on the UPEC virulence features may be due to their distinct expression patterns under certain environments and their distinct localization within *E. coli* cells. It is known that glutamate upregulates the levels of MepM, but not MepS, while aromatic amino acids upregulates the levels of MepS, but not MepM [25], suggesting that the MepM- and MepS-conferred endopeptidase function may dominate in distinct environmental conditions, such as the distinct host environments at day 2 and 14 post-infections. In addition, MepM is located on the inner membrane, while MepS is located on the outer membrane [10]. The distinct localization of the endopeptidases may affect their physiological functions and thus determine their distinct contributions to the virulence feature of UPEC.

The decreased motility caused by deletion of *mepM* was responsible for the reduction in the recovery of *mepM* mutant during UTIs. Flagella-mediated motility contributes to dissemination, particularly ascension to the upper urinary tract, and persistence during UTIs [13, 26]. It has been shown that the UPEC strain CFT073 without motility is significantly attenuated in bladder and kidney colonization at 48 h post-infection [13]. However, in our study, the bacterial counts of Δ*mepM*-UTI89 and UTI89 in the bladders and kidneys showed no significant difference at 48 h post-infection (Fig. 5a). It might be due to the UTI89 strain without *mepM* caused a 22% reduction in motility while the UPEC CFT073 strain without *fliC* is aflagellate and nonmotile [18]. In addition, we found that Δ*mepM*-UTI89 showed significantly lower bacterial counts than UTI89 in bladders and kidneys at 14 days post-infection (Fig. 5b). The increased motility caused by overexpression of FlhDC improved the *mepM* mutant’s fitness in UTIs (Fig. 7b). These findings were consistent with studies by Wright et al. [26] that motility contributes to UPEC persistence in the bladder and kidney at 14 days post-infection.

MepM is required for the survival of UPEC in the mouse urine. In our study, the difference between the trans-complemented strain versus the mutant was low in comparison with the difference between the mutant versus wild type strain (Fig. 6c, d). It may be due to a metabolic burden imposed by the recombinant plasmid p*mepM*. It is known that manipulations of recombinant plasmid DNA impose metabolic burden that could change numerous host cell properties which could significantly reduce growth rate [27, 28]. This may be the reason why the complemented strain did not show significantly increased survival in the mouse urine at 2 h, while the wild type strain showed significant differences with the *mepM* mutant at 2 h. In addition, bacteria almost were eliminated after 6 h incubation (less than 1% of inoculation) in the mouse urine, while bacteria still grew in the human urine (data not shown). This may be due to different components between the mouse urine and the human urine. The factors contributed to defective survival caused by *mepM* deletion in the mouse urine will be further investigated.

MepM not only provides the space for incorporation of new PG chain but also generates disaccharide-tetrapeptide chains for interaction between the active BAM complex proteins and newly synthesized PG during cell division [9, 29]. BAM complex are critical for the biogenesis of integral outer membrane proteins [30]. These suggest that MepM might contribute to the organization of outer membrane and other surface structures. However, in our study, the structures or functions of surface fimbriae (P, S, and curli fimbriae), which are involved in host cell adherence [15], might not be influence by MepM deficiency. Because deletion of *mepM* did not decrease ability to associate to epithelial cells (Fig. 2a) as well as the *mepM* mutant did not exhibit different transcript levels of fimbriae genes (Fig. S1). Other altered surface components caused by *mepM* deletion remain to be determined.

MepM contains a predicted transmembrane domain, a LysM domain, and a LytM domain and is shown in membrane fraction [10], suggesting that MepM is membrane localization. The LysM domain is associated with PG binding [31] while the LytM domain is required for the PG endopeptidase activity [10]. The histidine residue at position 314 within LytM domain is required for the Zn^2+^ coordination which is responsible for PG endopeptidase activity of MepM [9]. Thus, the compound that interferes Zn^2+^ coordination or inhibits the PG endopeptidase activity of LytM domain may be a potential drug to block the MepM’s function.

## Conclusions

Our study highlights the essential role of the peptidoglycan endopeptidase MepM in maintaining the intact virulence of UPEC, making it a candidate target for novel anti-infection interventions. Considering the previous finding that another peptidoglycan peptidase, MepS, is also crucial for UPEC’s full virulence, blocking the function of these endopeptidases could be a potential antimicrobial strategy against bacterial infections. While current antimicrobial measures have focused on blocking glycan synthesis in the cell wall (e.g., β-lactams), our study proposes a candidate approach by inhibiting the breakage of peptide linkage between glycan strands, presenting new opportunities for combating infections.

## Methods

### Bacteria strains, plasmids, cell lines, and growth condition

*E. coli* strains and plasmids used in this study are listed in Table 1. Bacterial strains were grown at 37 °C in 2 ml Luria Bertani (LB) broth in glass culture tube for overnight (16 h) at 200 rpm unless otherwise described. The iron-limited medium is M9 minimal medium containing 100 µM 2, 2-bipyridyl (DIP) [32]. To select and maintain the plasmids in the bacterial strains, the antibiotics were added in the culture medium. The antibiotics and their respective concentrations used for selecting strains with antibiotic resistance (Table 1) were kanamycin (50 µg/ml), chloramphenicol (15 µg/ml), gentamicin (100 µg/ml), and ampicillin (100 µg/ml). All antibiotics were purchased from Sigma-Aldrich (St Louis, MO, USA). The information of the plasmid vectors used in the study are shown in Table 1.

The human bladder epithelial cell line 5637 (HTB-9) and the murine macrophage cell line RAW264.7 (TIB-71) were purchased from the American Type Culture Collection (ATCC; Manassas, VA, USA). The 5637 cells were cultured in RPMI-1640 medium (Gibco) containing 10% fetal bovine serum (FBS; Gibco). The RAW264.7 cells were grown in DMEM medium (Gibco) supplemented with 10% FBS (Gibco). All cell lines were incubated at 37 °C in a humidified atmosphere of 5% CO_2_.

### Mutant and plasmid construction

The mutants of UTI89 were constructed by λ red mediated homologous recombination using PCR products containing sequences homologous to the targeting locus as described previously [33–35]. In brief, The PCR products containing kanamycin resistance cassettes flanked by approximately 38 bp of homology to the upstream and downstream regions of *mepM* gene was amplified from plasmid pKD4 using primers NK-*mepM*-F and NK-*mepM*-R shown in Table 2. These PCR products were transferred into UTI89 strains containing pKD46 by electroporation. Then, the resulting bacterial strains were selected on kanamycin plates. The primers ND*-mepM*-F and ND-*mepM*-R were used to distinguish the mutant and wild type strains, the expected amplified size of wild type strain and the mutant were 2530 bp and 3164 bp, respectively.

**Table 2:** Primers used in this study

Primers	Sequence (5’→3’)
***mepM*** **mutant construction**	
NK-*mepM*-F	TTGTCAAAACCATTGAGCTGGAACAGAACGAAATTCGTATAGGAATATCCTCCTTAGTTC
NK-*mepM*-R	CGACCATGACGAATAGCCACATAATAACCTGCTGCGCCTGTGTAGGCTGGAGCTGCTTCG
***mepM*** **mutant confirmation**	
ND-*mepM*-F	TCACGGCGATGATGATGATC
ND-*mepM*-R	CCCTGATTATGGAACATCGC
**p*mepM*** **construction**	
*mepM*-BamHI-F	TCACGGCGATTTCAACATGC
*mepM*-SacI-R	ATTCGAGCTCTTAATCAAACCGTAGCTGCG
**pCL-Cm construction**	
New-P1	TGTGTAGGCTGGAGCTGCTTCG
New-P2	ATAGGAATATCCTCCTTAGTTC
**pCL-FlhDC construction**	
pCL-*flhD*-HindIII-F	CGCCAAGCTTGTATCCATATGATGTTCCAGAT TATGCTGTGGGAATAATGCATACCTC
pCL-*flhC*-BamHI-R	ATCCTCTAGATTAGTGATGGTGATGGTGATGGTTCAGACCGACATATTTAAACTCG
**pmCherry construction**	
mCherry-NdeI-F	TATACATATGAGCAAGGGCGAGGAGGATAAC
mCherry-SalI-R	ACATGTCGACCTACTTGTACAGCTCGTC
pFPV25.1-SalI-F	GTAGGTCGACATGTCCAGACCTGCAGGCATG
pFPV25.1-NdeI-R	TGCTCATATGTATATCTCCTTCTTAAATC

To construct p*mepM*, the sequence encoding *mepM* was amplified from UTI89 genome by PCR with the primers *mepM*-BamHI-F and *mepM*-SacI-R. The PCR products were digested with BamHI and SacI and then cloned into the digested plasmid vector pCL1920.

To construct pCL-Cm, the DNA fragment containing the chloramphenicol-resistance cassette was generated from plasmid pKD3 [33] by PCR with the primers New-P1 and New-P2. The plasmid vector pCL1920 and the PCR product were digested with XbaI and then ligated to become pCL-Cm.

To construct pCL-FlhDC, the sequence encoding the N-terminally HA-tagged FlhD and C-terminally His_6_-tagged FlhC was amplified from the UTI89 genome by PCR with the primers pCL-*flhD*-HindIII-F and pCL-*flhC*-BamHI-R. After restriction digestion with HindIII and BamHI, pCL1920 and the PCR product were ligated to become pCL-FlhDC.

To construct pmCherry, the sequence encoding GFP of plasmid pFPV25.1 was replaced by a sequence encoding mCherry. The mCherry fragment was generated from plasmid pET mCherry LIC cloning vector (u-mCherry) (Addgene plasmid # 29,769) by PCR with the primers mCherry-NdeI-F and mCherry-SalI-R. Then, the plasmid backbone of pFPV25.1 was PCR amplified with the primers pFPV25.1-SalI-F and pFPV25.1-NdeI-R. After NdeI and SalI digestion, the two fragments were ligated to become pmCherry. The primers used for the plasmids construction are shown in Table 2. All restriction enzymes were purchased from NEB (Ipswich, MA, USA).

### Bacterial growth and survival assays

To study the growth of UPEC in LB and M9 and iron-limited media, overnight bacterial cultures were inoculated into 5 ml of the fresh media at 1:100 dilution and incubated at 37 °C with shaking at 200 rpm. The bacterial growth was determined by measuring the OD_600_ values of the cultures at the indicated time points.

To evaluate the survival of UPEC in mouse urine, pooled urine form 10 healthy mice was used. The overnight cultures of UTI89 and Δ*mepM*-UTI89 in a group, and Δ*mepM*-UTI89/pCL-Cm and Δ*mepM*-UTI89/p*mepM* in a group (1 × 10^7^ CFU/ml/strain) were washed with PBS, mixed, and inoculated into 50 µl of 90% mouse urine. The co-culture was incubated at 37 °C with shaking at 200 rpm. The live bacterial counts in the urine were determined at different time points. The strains in the urine culture were differentiated based on their distinct antibiotic resistance profiles.

### Uroepithelium association assay

The assay was performed as described previously [6, 36]. In brief, the bladder epithelial cell line 5637 cells were co-infected with equal amounts of UTI89 and Δ*mepM*-UTI89 (multiplicity of infection, MOI = 10) and incubated for 90 min. Afterward, the cells were washed three times with PBS and lysed by incubating with sterile water at 4 °C for 30 min. The lysates were then plated on LB plates supplemented with or without kanamycin (Km) to differentiate UTI89 (Km-sensitive strain) from Δ*mepM*-UTI89 (Km-resistant strain).

### Intracellular survival assay

The intracellular survival assay of UPEC was performed as described previously [6]. Briefly, the macrophage cell line, RAW264.7, was co-infected with two UPEC strains with a MOI of 10. After 30 min of incubation, the culture was treated with gentamicin (100 µg/ml) and incubated for 15 min to kill the bacteria that were not internalized by the macrophages. Then, the macrophages harboring UPEC were cultivated in the medium with a lower level of gentamycin (10 µg/ml). After 0, 2, 12, and 24 h of incubation, the medium of the culture was removed and the UPEC-harboring cells were lysed by addition of 0.1% (w/v) sodium deoxycholate in PBS. The number of surviving bacteria was determined as CFU by plating on LB agar. The bacterial strains with the same macrophage culture were differentiated based on their distinct antibiotic resistant profiles.

### Flow chamber-based cell infection model

To investigate the morphological switch of UPEC following interaction with bladder epithelial cells in vitro, we employed a previously described flow chamber (FC) bladder infection model [6, 17]. In brief (the flowchart shown in Fig. S4), the human bladder epithelial cell line 5637 was cultivated to confluence on the bottom of a flow chamber measuring 0.15 cm (height) × 0.2 cm (width) × 2 cm (length) in RPMI medium containing 10% FBS supplemented with 1% penicillin and streptomycin (Pen-Strep; Invitrogen). The flow chamber was custom-made as previously described by Wu et al. [37]. Subsequently, the cells were exposed to a continuous flow using a sterile polyethylene catheter connected to a syringe pump (NE-300, New Era Pump Systems, Inc, USA) with a flow rate of 0.18 ml/h of Epilife medium (Invitrogen) supplemented with 1% human keratinocyte growth medium (HKGM) and 1% Pen-Strep for 24 h. Following an 1 h removal of antibiotics, GFP-expressing *E. coli* (4 × 10^7^ CFU) were gradually introduced into the cell culture, and incubated for 6 h to allow for *E. coli* invasion into the 5637 cells. Subsequently, gentamicin and amikacin (100 µg/ml) were added to the flow medium to eliminate extracellular bacteria. After a 2 h antibiotic treatment, the flow medium was replaced with human urine to simulate UPEC-uroepithelium interaction within the bladder. After incubation for 24 h, bacteria within the flow chamber were collected and subjected to fluorescence microscopy analysis (IX81, Olympus, Tokyo, Japan). The procedures for collecting human urine samples were performed as described previously [6] and approved by the Institutional Reviewer Board (IRB) of National Cheng Kung University Hospital, Tainan City, Taiwan (no. A-ER-107-403 and A-ER-106-481).

### Motility assay

The bacterial strains were inoculated into 0.3% agar plates using a stab technique and incubated at 37 °C for 8 h [21]. The diameter of motility was measured in triplicate experiments and reported as the mean ± standard deviation. The strains used for motility comparison in Fig. 4a were UTI89/pCL1920, Δ*mepM*-UTI89/pCL-Cm, and Δ*mepM*-UTI89/p*mepM*.

### The mouse model of urinary tract infections

The eight-week-old female C3H/HeN mice were purchased from National Laboratory Animal Center, NARLabs, Taiwan and maintained under specific-pathogen-free conditions at Laboratory Animal Center, College of Medicine, National Cheng Kung University, Taiwan. The co-challenge mouse model of UTI was performed following the protocols described in our previous studies, with minor adjustments [6, 21]. Briefly, the mice (*N* = 10 or 11 in one batch) were deep anaesthetized by intraperitoneal injection of Zoletil 50 (Virvac, France, 50 mg/kg body weight) and Xylazine (Rompun, Bayer, Germany, 6.5 mg/kg body weight). Equal amounts of two UPEC strains (5 × 10^7^ CFU/strain) were mixed and transurethrally inoculated with a 50-µl bacterial suspension into the anaesthetized mice. The paired UPEC strains (UTI89 vs. Δ*mepM*-UTI89) used in the co-infection experiments shown in Figs. 5a, b, and 6a. The paired UPEC strains shown in Figs. 5c and 6b were Δ*mepM*-UTI89/pCL-Cm vs. Δ*mepM*-UTI89/p*mepM*, while the paired UPEC strains shown in Fig. 7b were Δ*mepM*-UTI89/pCL-Cm vs. Δ*mepM*-UTI89/pCL-FlhDC. Urine samples were collected daily after infection. The mice were euthanasia at either 2 or 14 days post-infection by using deep anesthesia as above mention, followed by carbon dioxide (CO_2_) inhalation. CO_2_ inhalation were performed by exposure to CO_2_ with a fill rate of 30–70% of the cage volume per minute, maintaining CO_2_ flow for at least five minutes after respiration ceases. The bladders and kidneys were collected, weighed, and homogenized in sterile culture tubes containing 3 ml of normal saline. The bacterial loads in the bladders and kidneys were determined by plating the homogenates onto LB agar plates containing appropriate antimicrobials. The UPEC strains within the tissues were differentiated based on their specific antibiotic resistance profiles.

To examine the morphological switch of UPEC in the mouse model of UTIs, mice were transurethrally inoculated with UPEC strains expressing GFP or mCherry fluorescence (5 × 10^7^ CFU) [6]. Urine samples were collected at indicated time points and fixed with 10% paraformaldehyde [38]. Then, the bacterial morphology in the urine was then examined using fluorescence microscopy (Zeiss Axio Observer Z1 inverted microscope, Carl Zeiss, Jena, Germany). After experiments, the mice were euthanasia as above mention. Animal studies were carried out according to the guideline by Council of Agriculture Executive Yuan Guideline for the Care and Use of Laboratory Animals, Republic of China. All of the animal experimental procedures were reviewed and approved by the Institutional Animal Care and Use Committee (IACUC) of National Cheng Kung University, Tainan City, Taiwan (approval number: 108,130).

### Western blot analysis

The western blot analysis followed the procedure was outlined by previous description [6]. Equal amount of total bacterial lysate were undergone separation via SDS-PAGE and subsequently transferred to PVDF membranes (Pall Corporation). For the detection of FliC proteins, rabbit polyclonal antisera targeting FliC (anti-H7, Becton Dickinson, Sparks, MD, United States) served as the primary antibody at a 1:10,000 dilution. The goat anti-rabbit horseradish peroxidase (HRP)-conjugated immunoglobulin G (IgG) antibodies (at a 1:10,000 dilution; KPL, Gaithersburg, MD) served as the secondary antibody. In addition, the mouse antiserum against OmpA, was used as primary antibodies at a 1:10,000 dilution. Subsequently, goat anti-mouse HRP-conjugated IgG antibodies (at a 1:10,000 dilution; KPL, Gaithersburg, MD) were employed as the secondary antibodies for detection of the OmpA proteins.

### Statistical analysis

The paired two-tailed student’s t-test was used in the statistical analysis of experiments in growth in urine, intracellular survival of macrophage, and binding of uroepitheliums, while the unpaired two-tailed student’s t-test was used in the statistical analysis of experiments in growth in LB, M9 and iron-limited media, morphological switch in vitro and in vivo, and motility assay [39]. The animal UTIs experiments were analyzed by using a nonparametric Wilcoxon matched-pair test [21, 40]. Significant difference of the bacterial counts in urine from infected animals was calculated by using two-way ANOVA with multiple-comparison test [41].

### Electronic supplementary material

Below is the link to the electronic supplementary material.

Supplementary Material 1

Supplementary Material 2

Supplementary Material 3

Supplementary Material 4

## Data Availability

All data and materials are fully available and are shown within the manuscript and its supplementary information file.

## Abbreviations

UPEC: Uropathogenic *Escherichia coli*
UTIs: Urinary tract infections
PG: Peptidoglycan
LB: Luria Bertani
h: Hour
rpm: Revolution(s) per minute
DIP: 2, 2-bipyridyl
PCR: Polymerase chain reaction
CFU: Colony-forming unit
PBS: Phosphate buffered saline
MOI: Multiplicity of infection
Km: Kanamycin
FC: Flow chamber
IRB: Institutional Reviewer Board
IACUC: Institutional Animal Care and Use Committee
Cm: Chloramphenicol
OM: Outer membrane
IM: Inner membrane
FBS: Fetal bovine serum
ATCC: American Type Culture Collection
CO_2_: Carbon dioxide
