# Supplementary material for: Peptidoglycan endopeptidase MepM of uropathogenic Escherichia coli contributes to competitive fitness during urinary tract infections
Source: BMC Microbiol. 2024 May 30;24:190. doi: 10.1186/s12866-024-03290-9 (PMC11137974; doi:10.1186/s12866-024-03290-9)
Supplement: Supplementary file 1 — Supplementary Material 1 [file 12866_2024_3290_MOESM1_ESM.pdf]

Fig. S1 The transcriptional levels of fimbriae- and K1 capsule-associated genes.

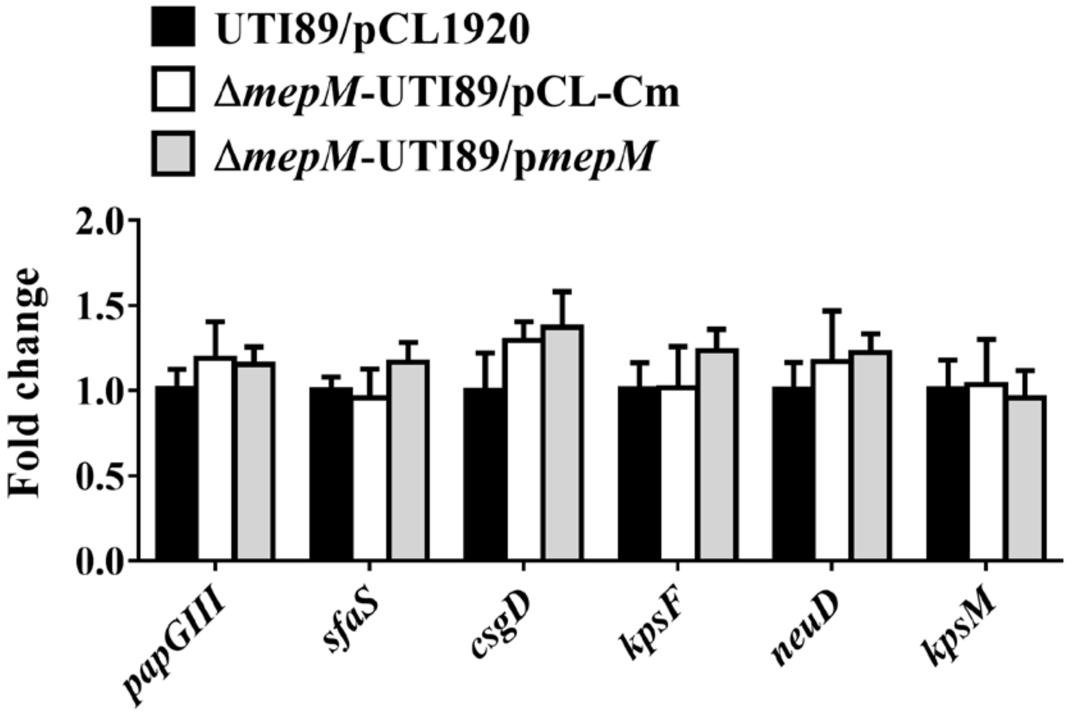

Impact of *mepM* deletion on the transcriptional levels of fimbriae- and K1 capsule-associated genes. The levels of genes *papGIII*, *sfaS*, and *csgD* reflected the transcript levels of the P fimbriae, S fimbriae, and curli fimbriae, respectively. The levels of genes *kpsF*, *neuD*, and *kpsM* represented the transcript levels of K1 capsule. The transcript levels of genes were determined by real-time PCR (qPCR). The transcript levels of the genes in each strain, which were normalized to those of the housekeeping gene *ftsZ*, were presented as the relative levels compared to those of UTI89/pCL1920. The results were derived from experiments in triplicate and are shown as the means ± standard deviations.

Primers used in real-time PCR analysis in Fig. S1.

Primers	Sequence (5'→3')
ftsZ-RT-F	CAATGGAACTTACCAATGAC
ftsZ-RT-R	TGTTTTACGCAGCGCTTGTG
papGIII-RT-F	GGCCTGCAATGGATTTACCTGG
papGIII-RT-R	CCACCAAATGACCATGCCAGAC
sfaS-RT-F	GGATGTTTCTTTGGGTAATCTG
sfaS-RT-R	CATTCCCTGTATTCGCATAG
csgD-RT-F	ATAACGAGATCGCTCGTTCG
csgD-RT-R	TGAGGTTATCGTTTGCCCAG
kpsF-RT-F	ATGTCGGTCGTAAAATGTC
kpsF-RT-R	GAACTAGCTTGAGGATTTCA
neuD-RT-F	GGTGCGGGTGGTTTTTCAAA
neuD-RT-R	ATGGTTTGCCTATCCCAATA
kpsM-RT-F	ACCGCACTATGCCAGACATC
kpsM-RT-R	GATCGTATCGATGGGTTTTA
