# Supplementary material for: Peptidoglycan endopeptidase MepM of uropathogenic Escherichia coli contributes to competitive fitness during urinary tract infections
Source: BMC Microbiol. 2024 May 30;24:190. doi: 10.1186/s12866-024-03290-9 (PMC11137974; doi:10.1186/s12866-024-03290-9)
Supplement: Supplementary file 2 — Supplementary Material 2 [file 12866_2024_3290_MOESM2_ESM.pdf]

Fig. S2 Capsule stain images of UTI89 and Δ*mepM*-UTI89.

(A) UTI89

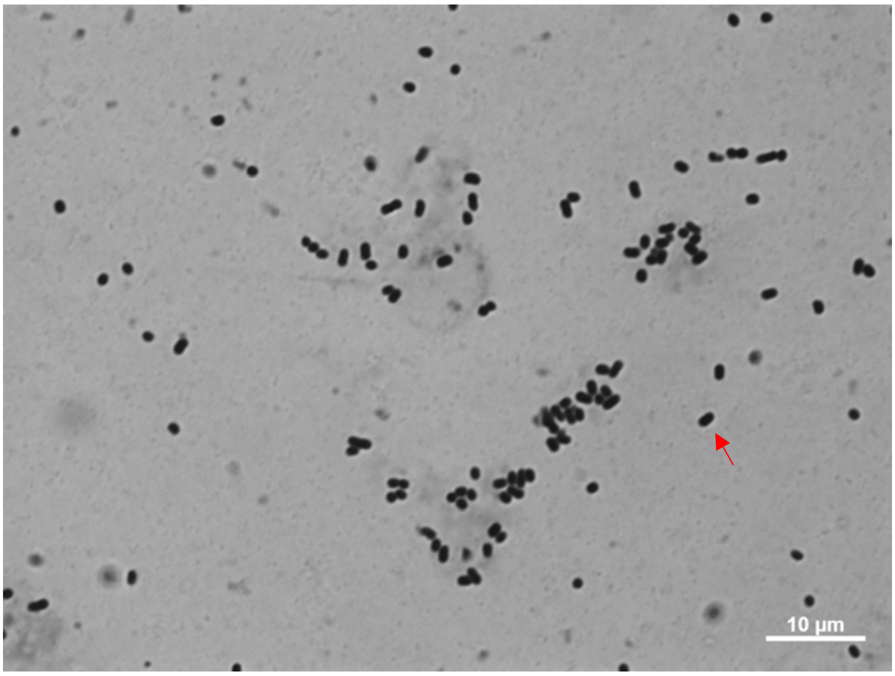

(B) Δ*mepM*-UTI89

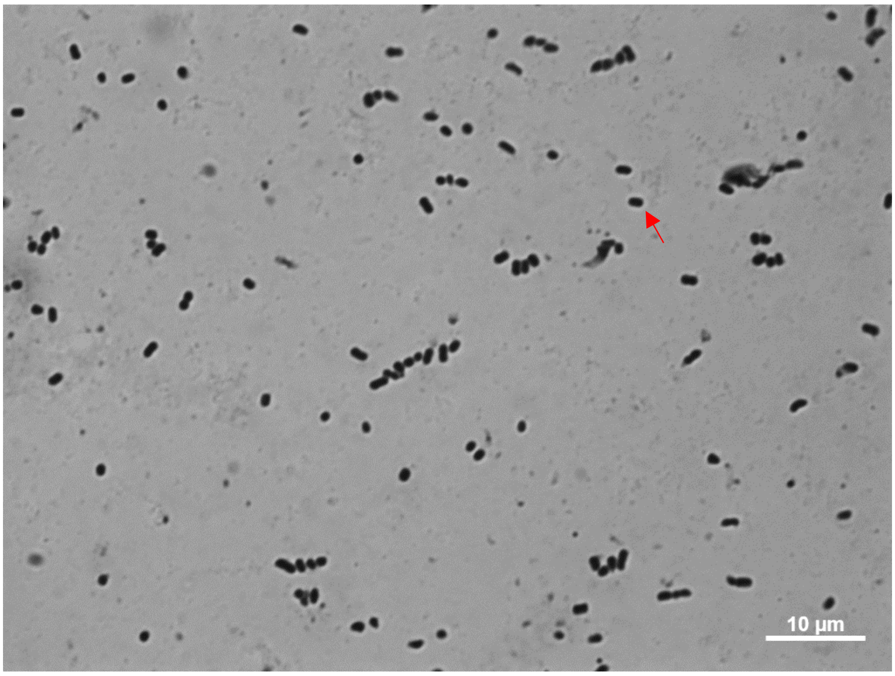

Encapsulated UTI89 and Δ*mepM*-UTI89 stained using Anthony's capsule staining method [1]. Bright-field microscopy images of the strains at 1,000X magnification. The red arrows indicate clear halo (capsule) around the rod-shaped bacterium.

Reference

1. Anthony EE, Jr.: A Note on Capsule Staining. *Science* 1931, 73(1890):319-320.
