# Supplementary material for: Peptidoglycan endopeptidase MepM of uropathogenic Escherichia coli contributes to competitive fitness during urinary tract infections
Source: BMC Microbiol. 2024 May 30;24:190. doi: 10.1186/s12866-024-03290-9 (PMC11137974; doi:10.1186/s12866-024-03290-9)
Supplement: Supplementary file 3 — Supplementary Material 3 [file 12866_2024_3290_MOESM3_ESM.pdf]

Fig. S3 The original and unprocessed blot images of Fig. 4b.

Upper panel: The rabbit anti-FliC antiserum (Becton Dickinson, Sparks, MD, USA)

(A) Individual images of film and PVDF membrane

1 2 3 4 5 6 7 8 9 10

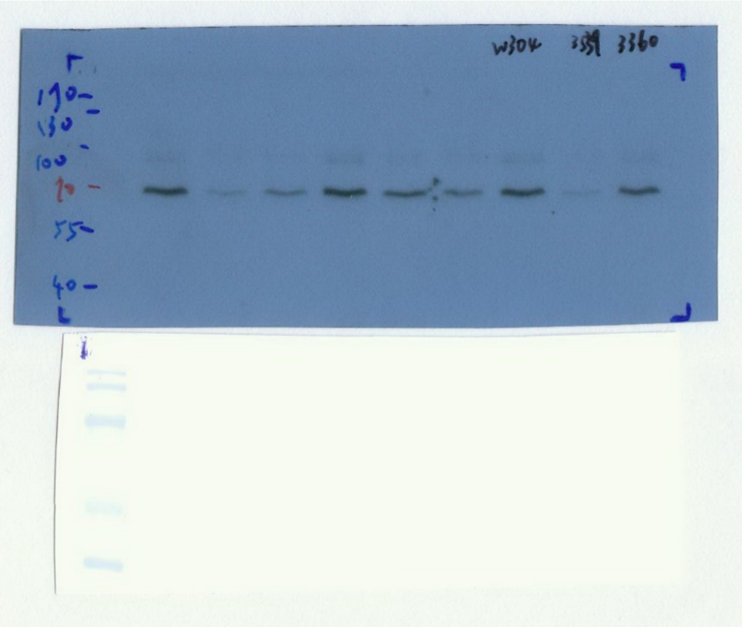

(B) Grayscale image of (A)

1 2 3 4 5 6 7 8 9 10

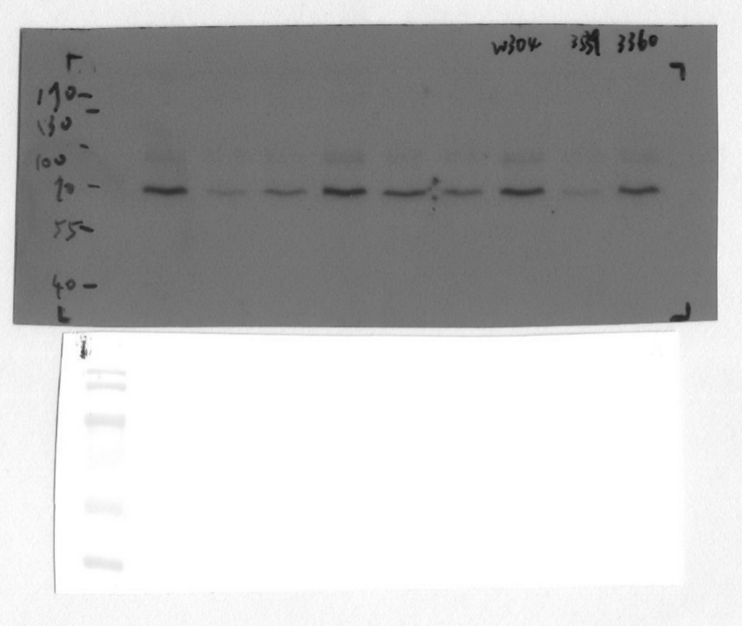

(C) The merged image of film and PVDF membrane

1 2 3 4 5 6 7 8 9 10

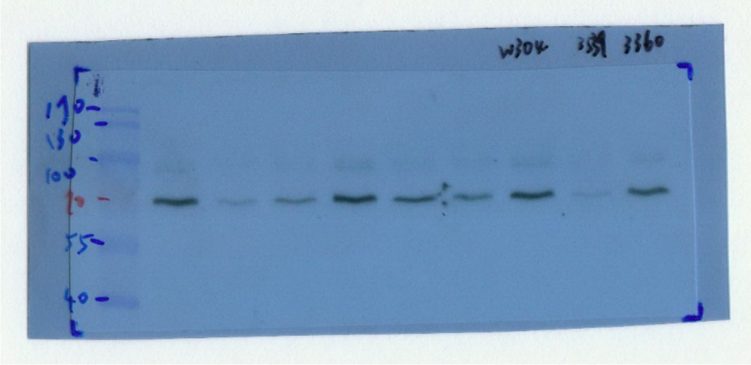

(D) Grayscale image of (C)

1 2 3 4 5 6 7 8 9 10

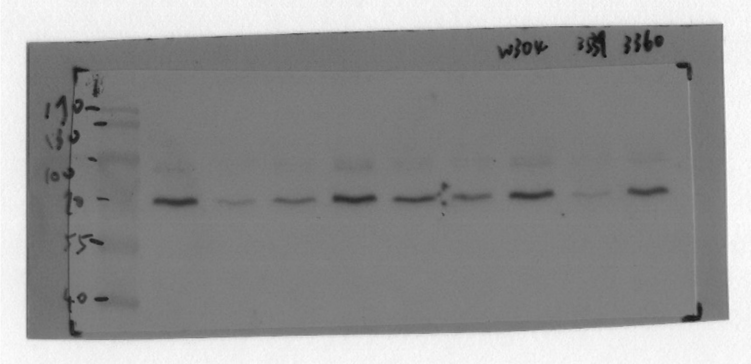

Lane 1: Protein molecular weight markers

Lane 8: UTI89/pCL1920

Lane 9: Δ*mepM*-UTI89/pCL-Cm

Lane 10: Δ*mepM*-UTI89/p*mepM*

Lower panel: The mouse anti-OmpA antiserum

(E) Individual images of film and PVDF membrane

1 2 3 4 5 6 7 8 9 10

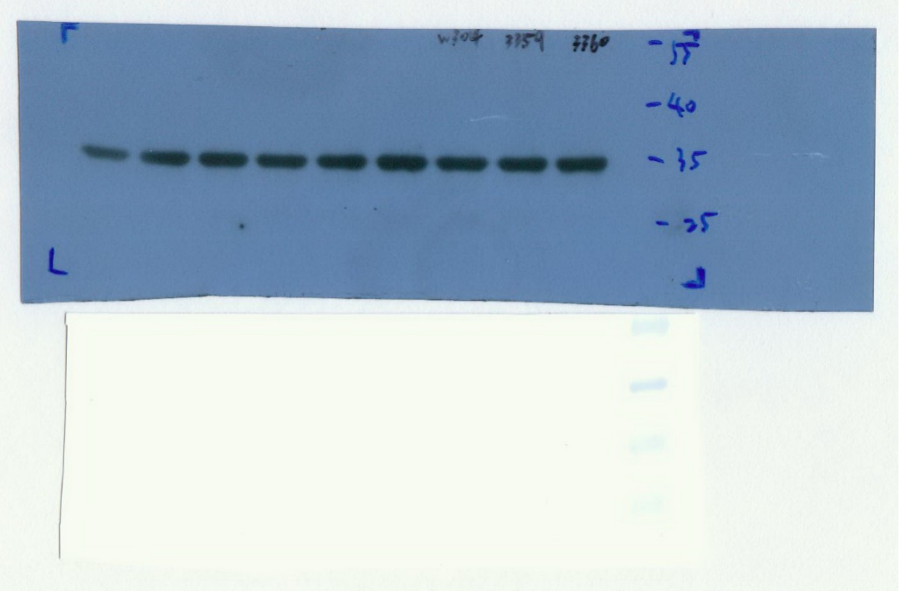

(F) Grayscale image of (E)

1 2 3 4 5 6 7 8 9 10

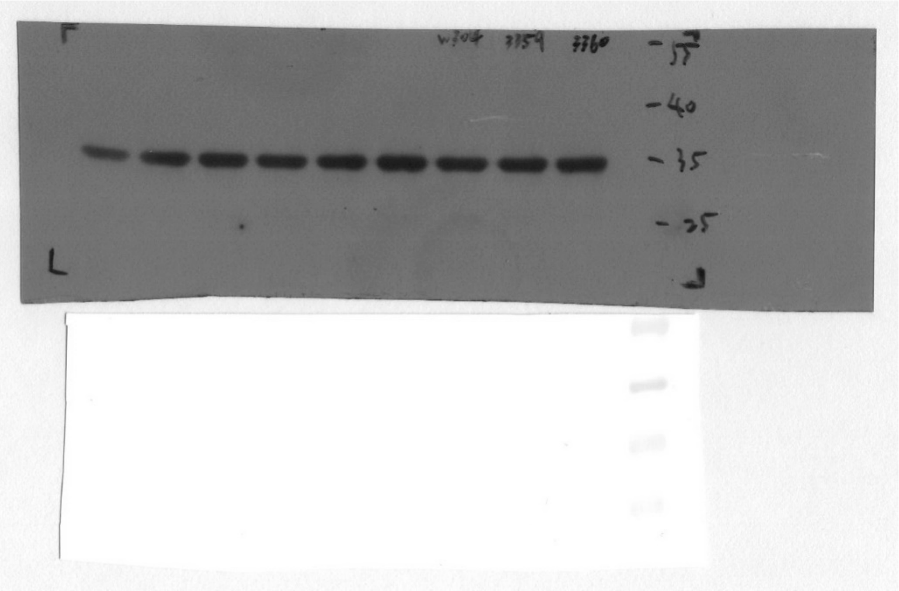

(G) The merged image of film and PVDF membrane

1 2 3 4 5 6 7 8 9 10

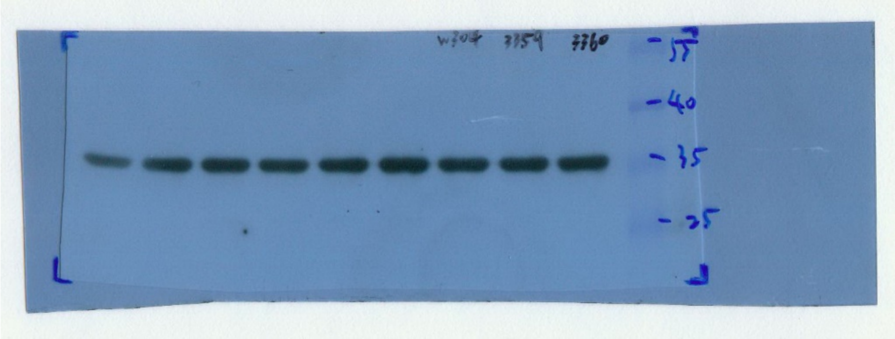

(H) Grayscale image of (G)

1 2 3 4 5 6 7 8 9 10

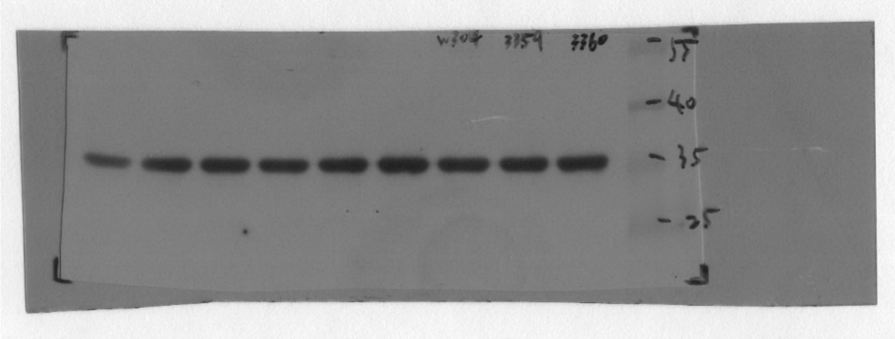

Lane 7: UTI89/pCL1920

Lane 8: Δ*mepM*-UTI89/pCL-Cm

Lane 9: Δ*mepM*-UTI89/p*mepM*

Lane 10: Protein molecular weight markers
