# Supplementary material for: Peptidoglycan endopeptidase MepM of uropathogenic Escherichia coli contributes to competitive fitness during urinary tract infections
Source: BMC Microbiol. 2024 May 30;24:190. doi: 10.1186/s12866-024-03290-9 (PMC11137974; doi:10.1186/s12866-024-03290-9)

Fig. S4 The flowchart of flow chamber-based cell infection model

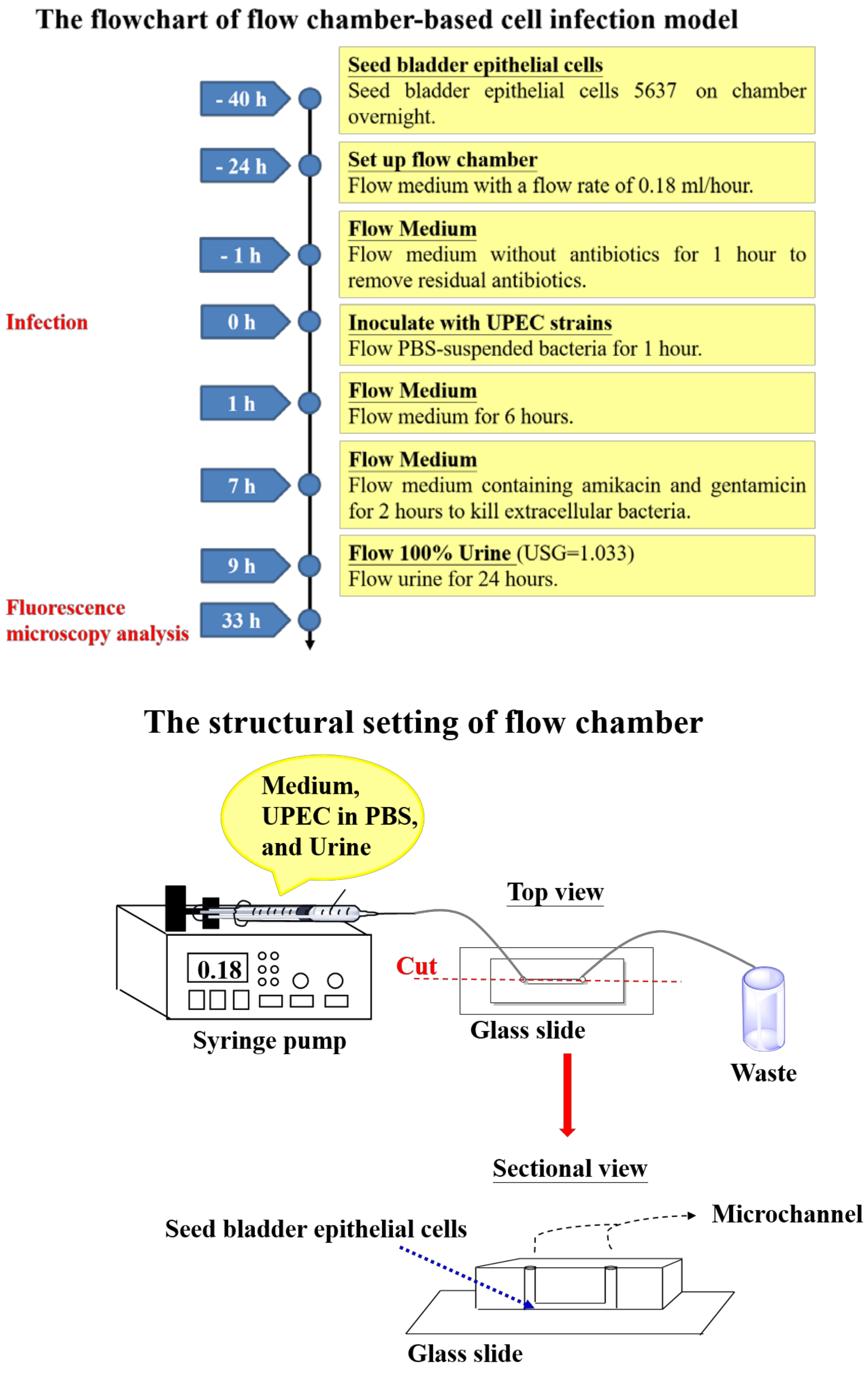

Supplement: Supplementary file 4 — Supplementary Material 4 [file 12866_2024_3290_MOESM4_ESM.pdf]
